# Early life socioeconomic adversity is associated in adult life with chronic inflammation, carotid atherosclerosis, poorer lung function and decreased cognitive performance: a cross-sectional, population-based study

**DOI:** 10.1186/1471-2458-11-42

**Published:** 2011-01-17

**Authors:** Chris J Packard, Vladimir Bezlyak, Jennifer S McLean, G David Batty, Ian Ford, Harry Burns, Jonathan Cavanagh, Kevin A Deans, Marion Henderson, Agnes McGinty, Keith Millar, Naveed Sattar, Paul G Shiels, Yoga N Velupillai, Carol Tannahill

**Affiliations:** 1 Glasgow Clinical Research Facility, Tennent Building, 38 Church Street, Western Infirmary, Glasgow G11 6NT, UK; 2 Robertson Centre for Biostatistics, University of Glasgow, Level 11, Boyd Orr Building, University Avenue, Glasgow G12 8QQ, UK; 3 Glasgow Centre for Population Health, 1st Floor, House 6, 94 Elmbank Street, Glasgow G2 4DL, UK; 4 Medical Research Council Social and Public Health Sciences Unit, 4 Lilybank Gardens, Glasgow G12 8RZ; Centre for Cognitive Ageing & Cognitive Epidemiology, University of Edinburgh, Edinburgh, UK; The George Institute for International Health, Sydney, Australia; 5 Scottish Government, St. Andrew's House, Regent Road, Edinburgh EH1 3DG, UK; 6 College of Medical, Veterinary and Life Sciences, University of Glasgow: Psychological Medicine, Gartnavel Royal Hospital, 1055 Great Western Road, Glasgow G12 0XH, UK; 7 NHS Greater Glasgow & Clyde, Glasgow Royal Infirmary, Department of Clinical Biochemistry, Macewen Building, 84 Castle Street, Glasgow G4 0SF; Department of Clinical Biochemistry, First Floor, Link Building, Aberdeen Royal Infirmary, Foresterhill, Aberdeen AB25 2ZD, UK; 8 Medical Research Council Social and Public Health Sciences Unit, 4 Lilybank Gardens, Glasgow G12 8RZ, UK; 9 University of Glasgow, Division of Cardiovascular and Medical Sciences, based at Vascular Biochemistry, 4th Floor, Queen Elizabeth Building, Glasgow Royal Infirmary, 10 Alexandra Parade, Glasgow G31 2E, UK; 10 University of Glasgow, Faculty of Medicine, University Department of Surgery, Level 2, Queen Elizabeth Building, Glasgow Royal Infirmary, 10 Alexandra Parade, Glasgow G31 2ER, UK

## Abstract

**Background:**

Socioeconomic gradients in health persist despite public health campaigns and improvements in healthcare. The Psychosocial and Biological Determinants of Ill-health (pSoBid) study was designed to uncover novel biomarkers of chronic disease that may help explain pathways between socioeconomic adversity and poorer physical and mental health.

**Methods:**

We examined links between indicators of early life adversity, possible intermediary phenotypes, and markers of ill health in adult subjects (n = 666) recruited from affluent and deprived areas. Classical and novel risk factors for chronic disease (lung function and atherosclerosis) and for cognitive performance were assessed, and associations sought with early life variables including conditions in the parental home, family size and leg length.

**Results:**

Associations were observed between father's occupation, childhood home status (owner-occupier; overcrowding) and biomarkers of chronic inflammation and endothelial activation in adults (C reactive protein, interleukin 6, intercellular adhesion molecule; *P *< 0.0001) but not number of siblings and leg length. Lung function (forced expiratory volume in 1 second) and cognition (Choice Reaction Time, the Stroop test, Auditory Verbal Learning Test) were likewise related to early life conditions (*P *< 0.001). In multivariate models inclusion of inflammatory variables reduced the impact and independence of early life conditions on lung function and measures of cognitive ability. Including variables of adult socioeconomic status attenuated the early life associations with disease biomarkers.

**Conclusions:**

Adverse levels of biomarkers of ill health in adults appear to be influenced by father's occupation and childhood home conditions. Chronic inflammation and endothelial activation may in part act as intermediary phenotypes in this complex relationship. Reducing the 'health divide' requires that these life course determinants are taken into account.

## Background

Socioeconomic gradients in health are widely observed even in developed countries, but are not yet fully explained [1-8]. It is increasingly accepted that variation in the prevalence of classical risk factors for chronic diseases (smoking, blood pressure etc) only partially accounts for these social class gradients (for example in coronary heart disease (CHD)) [9-12] and that there is a need to uncover other potential explanatory pathophysiological mechanisms. Possible candidates are chronic 'low grade' activation of the innate immune system [13,14] (which may start early in life [15-17] and be influenced by cumulative effects of socioeconomic status over the life course [18]), insulin resistance and endothelial dysfunction [19,20]. Chronic inflammation is believed to be a contributory aetiological factor in a range of conditions including atherosclerosis, lung disease and decreased cognitive performance and dementia [21-23].

Increasing evidence indicates that socioeconomic circumstances during the early years of life are important determinants of health outcomes in adulthood, with the propensity for poor health in adulthood being greatest among those from disadvantaged backgrounds. Risk of mortality accumulates during the life course [24,25] and exposure to risk factors can occur many years before the development of an outcome [26]. Adverse childhood socioeconomic position has been reported to be associated with a poorer health profile in mid adulthood (45 years of age), independent of adult social position and across diverse measures of disease risk and physical and mental functioning [24]. At mid adulthood, associations with childhood social class were identified for blood pressure, body mass index, high density lipoprotein, triglycerides, lung function, depressive symptoms and chronic widespread pain. Increased risk of ill-health was related to participants' father's occupation i.e. from class I (professional occupations) to V (unskilled occupations).

Whether increased morbidity and mortality in adulthood are the result of biological programming due to critical events *in utero*, the accumulation and interaction of harmful exposures along the pathway between infancy and adulthood, or a combination of both remains unclear for most diseases. It follows that better understanding of the antecedents of the greater burden of chronic disease and disability in relatively deprived populations gained from an exploration of life course effects from pre-birth [27] through childhood [26,28-33] to adult life is essential to tackle the growing 'health divide'.

Glasgow, Scotland has a population that exhibits marked gradients in physical and mental health: at postcode sector level (average population 3000-5000 individuals), the difference in male life expectancy between the most and least deprived areas is 28.7 years [34]. Between these areas there is a 7-fold variation in levels of CHD and stroke mortality, a 5-fold range in incidence of diabetes, and differences of a similar magnitude in psychiatric-related hospital admission rates [35]. The present work relates indices of childhood living conditions to biomarkers of chronic inflammation (C reactive protein, interleukin 6), insulin resistance (elevated circulating levels of insulin and glucose), endothelial activation (a dysfunctional state where there is increased expression of cellular adhesion molecules and other factors), and to indicators of atherosclerosis (carotid artery intima-media thickness (cIMT), carotid artery plaque count), cognitive performance, and lung function in a group of adult subjects drawn from affluent and deprived areas of Glasgow. The overall aim of the study was to explore possible links between adverse early life conditions, intermediary phenotypes such as a persistent chronic inflammatory state and increased insulin resistance and endothelial activation and health outcomes in adulthood (atherosclerosis, lung function and cognitive impairment), which may share common aetiological determinants [19-23]. More specifically, we sought evidence of associations of adverse early life conditions (a) with biomarkers of inflammation and endothelial dysfunction, and (b) with indices of atherosclerosis, lung function and cognitive ability in adulthood. We then tested the extent to which the statistical relationships between adverse childhood conditions and health outcomes in adulthood were attenuated when the associations with biomarkers and adult socioeconomic factors were included in the model.

## Methods

### Ethical approval

The study was approved by the Glasgow Royal Infirmary Research Ethics Committee and all participants gave written informed consent.

### Study population and protocol

The design - including recruitment strategy, response rates and study protocol - of the psychological, social and biological determinants of ill health (pSoBid) study has been described in detail elsewhere [36,37]. Briefly, selection of subjects was based on the Scottish Index of Multiple Deprivation (SIMD) [38] which ranks small areas of Scotland on the basis of multiple deprivation indicators (multiple indicators across 6 domains, namely: income (e.g. number of adults and children in Income Support households); employment (e.g. unemployment claimant count average over 12 months, number of working age Incapacity Benefit recipients); health (e.g. number of hospital episodes related to alcohol use and drug use, number of hospital emergency admissions); education, skills and training (e.g. number of working age people with no qualifications, number of school leavers age 16 + not in education); geographic access and telecommunications (e.g. drive time access to GP, supermarket and primary school); and housing (e.g. number of persons in households which are overcrowded, number of persons in households which are without central heating)), allowing identification of the least and most deprived areas in the Greater Glasgow and Clyde Health Board area. Five general practices with the highest percentage of patients aged 35-64 years living in areas classified as being in the bottom 5% of SIMD (i.e. relatively deprived) areas agreed to participate as did a further five practices with the highest percentage of patients aged 35-64 years living in areas classified as being in the top 20% of the SIMD (i.e. relatively affluent). At the time of sampling 31.4% of the Glasgow population resided in the bottom 5% of the 2004 SIMD and 6% resided in the top 20% (only 1.4% of the Glasgow population resided in the top 5% of the 2004 SIMD). The Health Information and Technology section of the Health Board generated a target random population of 21 672 people from the practice lists of these ten practices and 12 groups of 300 participants were selected according to strata defined by the combination of SIMD classification, gender and age group (35 to 44, 45 to 54 and 55 to 64 years), giving a total sampling frame of 3,600 subjects. As the sampling frame was constructed from general practice lists, this included individuals regardless of whether or not they actually visited their general practitioner. As the study progressed, over-sampling of subjects from the most deprived group was required (due to the lower response rate) and the Health Information and Technology section was approached to select randomly further potential subjects from the target population. We recruited approximately equal numbers from most and least deprived areas, equal numbers of males and females and equal numbers from each age group (35-44, 45-54 and 55-64 years). As described in the design paper [36] we were able to interrogate the information held by computer systems in the general practices and extract anonymously key characteristics of the subjects who responded to the invitation (and became participants) and those who declined or did not respond ('non-participants'). Participants and non-participants by design fell into the same age and sex categories. Reliable information was available on their smoking status and use of prescription drugs. A comparison of the two groups (666 participants; 1654 non participants) showed mostly modest but in some cases statistically significant differences (Appendix 1; see Additional file 1). The most notable difference was a higher percentage of participants compared to non-participants on prescription drugs, in the most deprived group.

Participants attended for two visits (around 2 weeks apart) between December 2005 and May 2007. In visit 1, they completed lifestyle and psychological questionnaires and underwent measurement of blood pressure, heart rate, hip, waist and mid-thigh circumference and assessment of lung function (measured by Forced Expiratory Volume in one second (FEV1)). The lifestyle questionnaire included questions on physical activity, alcohol intake, dietary habits and smoking behaviour. At visit 2, participants attended fasting, for blood to be taken for biochemical analyses. Height and weight were measured. After being provided with breakfast, subjects completed cognitive tests as outlined below. Finally, carotid artery ultrasound was performed.

### Early life and adult individual level socioeconomic status

A number of indices based on participant recall were used to assess childhood conditions at age 11 years. These were: number of siblings, whether or not their parents owned their home, father's occupational category, whether or not they reported being bullied as a child, whether or not their parents owned a car, overcrowding (number of occupants in house divided by number of rooms), leg length (a surrogate measure of nutrition during growth [31-33]) and trunk length. Father's occupational category was classified using the Registrar General's Social Class Classification (that is: I - professional occupations; II - managerial and technical occupations; IIINM - skilled occupations (non-manual); IIIM - skilled occupations (manual); IV - partly skilled occupations; and V - unskilled occupations). For the purposes of analysis, we merged non-manual social classes (I, II and IIINM) and compared them with merged manual social classes (IIIM, IV and V). Current (i.e. adult) socioeconomic status was assessed from income (average household income in £), educational achievement (years in education), and home ownership (owner occupier, tenant - local authority, tenant - private, living with parents, other) [5,20].

### Carotid artery ultrasound analysis

All scans were performed on a Siemens Acuson Sequoia 512 scanner with an L7 5-12 MHz linear array broadband transducer (Siemens Medical Solutions, Erlangen, Germany) using techniques described elsewhere [37]. Briefly, scans were analysed using the eTrack software provided by the Department of Vascular Medicine and Physiology, Academic Medical Centre, Amsterdam, The Netherlands. All scans were analysed by the same reader, blinded to the identities of the participants. Carotid intima-media thickness (cIMT) was measured on the far wall of each arterial segment, averaged along a 1 cm length. Number of plaques per subject was counted, with plaque being defined as a focal structure encroaching into the arterial lumen of at least 0.5 mm or 50% of the surrounding IMT value, or demonstrating a thickness >1.5 mm as measured from media-adventitia interface to intima-lumen interface [39]. Reader reproducibility was assessed by repeat reading of a proportion of the scans, and was consistently within the predefined certification limits of a coefficient of variation of < 5%.

### Biochemical analysis

All blood samples were separated and frozen at -80°C within 1 hour of venepuncture, except for samples for cholesterol, triglycerides, low density lipoprotein (LDL), high density lipoprotein (HDL), high sensitivity C-reactive protein (CRP) and glucose, which were processed immediately. Cholesterol and triglyceride were determined by enzymatic colorimetric assays on a Roche 917 analyser (Roche Diagnostics Ltd., Burgess Hill, United Kingdom). Lipid fractions were measured using ultracentrifugation and precipitation methods [40]. Glucose was measured by hexokinase/glucose-6-phosphate dehydrogenase assay on an Abbott c8000 analyser (Abbott Diagnostics, Maidenhead, United Kingdom). Insulin was measured by a sandwich Enzyme-Linked Immunosorbent Assay (ELISA) (Mercodia AB, Uppsala, Sweden). High sensitivity C-reactive protein (CRP) was measured by an immunoturbidimetric assay (Roche Diagnostics Ltd., Burgess Hill, United Kingdom). Interleukin-6 (IL-6) and Intercellular Adhesion Molecule-1 (ICAM) were measured by sandwich ELISA (R&D Systems Europe Ltd., Abingdon, United Kingdom). von Willebrand Factor (vWF) was measured using an in-house ELISA, employing rabbit anti-human polyclonal antibodies (DAKO plc, High Wycombe, United Kingdom).

### Cognitive Function Tests

A series of tests was employed to assess the principal cognitive domains of executive function, reaction and decision processing, and memory. The number and duration of the tests were constrained by the time demands that might reasonably be made upon participants who were required to attend the research clinic on two separate occasions.

The tests employed were *(a) for Executive function: *the Stroop Colour-Word Task [41] which assesses ability to inhibit dominant and over-learned responses (test result is the number of correct responses on the colour-word interference component in a set time); *(b) for Reaction and Decision Processing*: the Choice Reaction Time test (CRT) in which the reaction time was decomposed into encoding ("thinking") time and response ("movement") time and measured in milliseconds by a computerised system [42] and is sensitive to a range of factors affecting motor and decision speed [43]; and *(c) for Memory: *the Auditory Verbal Learning Test (AVLT), which assesses speed of learning, recall and recognition performance [44].

### Statistical analysis

Continuous variables are summarised as mean and standard deviation (SD) or geometric mean, depending on their distribution; categorical variables are summarised as frequencies and percentages. For comparisons between deprivation groups, linear or logistic regression was used with adjustment for age and sex.

Associations between markers of early life and adult socioeconomic status were assessed using linear or logistic regression models, reported in terms of the R^2 ^or Nagelkerke generalised R^2 ^statistics and levels of statistical significance.

A series of regression models was used to investigate outcome measures of *(a) *biomarkers of chronic inflammation and endothelial dysfunction and *(b) *adult lung function, cognitive performance and carotid atherosclerosis. A base model adjusted for age and sex only (Model 0) was constructed. Terms for adverse early life conditions were then included (Model 1). Variables reflecting chronic inflammation/endothelial activation were then added (Model 2) to assess how this affected the associations seen in Model 1. A final model (Model 3) included those terms in Model 1 plus adult markers of socioeconomic status to assess whether early life variables were still independently associated with an outcome. For linear regression models, selected outcomes were log-transformed prior to analysis; the Tables report the regression coefficients with 95% confidence intervals (CIs) and levels of significance. For binary outcome measures, effect estimates are reported as odds ratios. An F-test was used to assess the impact of inclusion of early life variables on the goodness of fit of Models 1 to 3.

Analyses were conducted in SAS v9.1 and R v2.8.

## Results

A total of 666 subjects were recruited to the study from 2,712 invited to participate (giving an overall response rate of 24.6%). By design there were approximately equal numbers of men and women in each of the three age decades; 342 were drawn from the least deprived areas and 324 from the most deprived. For the least deprived group as a whole the response rate was 33.9%, and for the most deprived group the response rate was 19.0%.

Table 1 provides summary statistics by area level deprivation (as defined by SIMD) for variables related to early life conditions, individual socioeconomic status (SES) as adults, biomarkers of chronic inflammation and endothelial dysfunction, CHD risk factors, carotid atherosclerosis, body habitus, lung function and cognitive performance. There were significant differences between groups in early life variables i.e. the number of siblings in the family, a measure of habitation overcrowding at age 11 (number of occupants in house divided by the number of rooms), father's occupational category, and whether or not parents owned the family home or a car. There was no significant difference between groups in relation to being bullied as a child. Height and trunk length but not weight differed by deprivation. Leg length (a surrogate measure of early life growth, possibly related to nutrition [31,32]) was significantly greater in the more affluent group. Individual level indices of socioeconomic status as an adult (household income, home ownership and years in education) varied as expected.

**Table 1:** Mean levels of early life conditions, biomarkers of chronic disease and cognitive function by area level deprivation category

	SIMD Least Deprived (n = 342)	SIMD Most Deprived (n = 324)	***P***^a^
***Early life conditions***			
Number of siblings	2.6(1.2)^b^	3.6(1.8)	< 0.0001
People/room	1.2(0.5)	1.8(0.9)	< 0.0001
Parents owned home	49.4%	5.9%	< 0.0001
Parents owned car	57.6%	19.6%	< 0.0001
Reported being bullied	24.6%	28.7%	0.24
Father's occupational category ^d ^(non-manual/manual)	59.6%/40.4%	14.6%/85.4%	< 0.0001
***Adult Socioeconomic status***			
Average household income	£41,699(11,921)	£16,461(10056)	< 0.0001
Years of education	16.1(3.6)	11.8(2.5)	< 0.0001
Current home status (owner-occupier/tenant)	97.7%/2.3%	29.9%/70.1%	< 0.0001
***Body Habitus***			
Height (cm)	171(9)	165(9)	< 0.0001
Weight (kg)	78.7(15.3)	78.2(18.4)	0.78
BMI (kg/m^2^)	26.9(4.5)	28.7(6.3)	< 0.0001
Leg-length (cm)	81.9(6.0)	78.7(5.4)	< 0.0001
Trunk-length (cm)	89.3(5.1)	86.5(5.1)	< 0.0001
***Inflammatory Biomarkers***			
CRP (mg/L)	2.10(2.73)^c^	3.56(3.91)	< 0.0001
IL-6 (pg/ml)	1.74(1.49)^c^	2.50(1.60)	< 0.0001
ICAM (ng/ml)	241.3(54.9)^c^	315.4(97.2)	< 0.0001
vWF (IU/dl)	129(39)	155(46)	< 0.0001
***Classical Risk factors***			
Cholesterol (mmol/l)	5.29(1.03)	4.95(1.05)	< 0.0001
HDL Cholesterol (mmol/l)	1.43(0.38)	1.30(0.39)	< 0.0001
Current Smoker (%)	6.3%	44.6%	< 0.0001
BP systolic/diastolic (mmHg)	135/81	136/81	0.48/0.74
***Insulin Resistance***			
HOMA-IR ^e^	1.52(1.22)	1.81(1.60)	0.012
***Carotid Atherosclerosis***			
Carotid IMT (mm)	0.68(0.12)	0.70(0.15)	0.014
Plaque present (% yes)	43.1%	58.3%	< 0.0001
***Cognitive Function***			
Stroop test (s)	103.0(15.1)	93.0(19.8)	< 0.0001
Choice Reaction Time (ms)^e^	531(101)^c^	630(185)	< 0.0001
AVLT (words recalled)^e^	12.4(1.9)	10.9(2.4)	< 0.0001
***Lung Function***			
FEV1 (L)^e^	3.2(0.8)	2.7(0.7)	< 0.0001
			

^a ^p-values from linear or logistic regression models, adjusted for age and sex

^b ^Values given are mean with 1 Standard Deviation in parenthesis for continuous variables; ^c ^Indicates use of geometric means;

^d ^Fathers occupational category for Least Deprived was unemployed for n = 1 (0.3%) and unknown/unclassifiable for n = 19

(5.6%); Father's occupational category for Most Deprived was unemployed for n = 10 (3.1%) and unknown/unclassifiable

for n = 33 (10.1%); P value derived by Chi squared across the distribution;

^e ^Homeostasis Model Assessment of Insulin Resistance (HOMA-IR); Auditory Verbal Learning Test (AVLT); Forced

Expiratory Volume (FEV1); Choice Reaction Time (CRT), data for the thinking time element of test presented.

Associations between early life conditions and indicators of adult socioeconomic status are given in Table 2. Overcrowding at age 11 years was related strongly to number of siblings (note this variable is used in the calculation of people per room), whether parents owned their home or a car, and father's occupational category. The dependency of early life conditions on adult SES indicators was low; R^2 ^was in the range 0.1 to 17.6%.

**Table 2:** Associations of indictors of early life conditions and adult socioeconomic status

	Number of siblings	People/room^a^	Parents owned house^b^	Parents owned car^b^	Reported being bullied^b^	Father's occup category^c^	Leg length	Household income	Years of Education	Current home status^d^
*Early life conditions*										
Number of siblings	-	29.0%***	4.0%***	1.3%*	0.0%	4.9%***	1.3%**	2.6%**	4.0%***	6.6%***
People/room^a^	29.0%***	-	34.0%***	19.3%***	0.4%	27.9%***	3.6%***	9.1%***	14.4%***	16.7%***
Parents owned home^b^	2.8%***	22.3%***	-	26.9%***	0.0%	31.3%***	2.5%***	5.3%***	13.8%***	17.6%***
Parents owned car^b^	0.9%*	13.9%***	28.5%***	-	0.0%	19.9%***	1.4%**	10.5%***	10.5%***	14.1%***
Reported being bullied^b^	1.0%*	0.4%	0.1%	0.0%	-	0.1%	0.6%	3.8%***	0.1%	2.8%**
Fathers occup category^c^	3.6%***	19.8%***	32.8%***	19.8%***	0.1%	-	1.4%**	6.5%***	15.6%***	12.7%***
Leg length	1.3%**	3.6%***	3.5%***	1.9%**	0.5%	1.9%**	-	1.0%*	4.9%***	2.0%**
*Adult socioeconomic status*										
Household income	2.6%***	9.1%***	8.2%***	14.0%***	0.0%	8.8%***	1.0%*	-	19.5%***	43.9%***
Years of Education	4.0%***	14.4%***	18.9%***	13.8%***	0.1%	20.4%***	4.9%***	19.5%***	-	31.3%***
Current home status^d^	4.7%***	12.3%***	18.3%***	13.9%***	0.1%	12.4%***	1.5%**	34.5%***	21.9%***	-

Variables in the rows are used to predict variables in the columns, using linear or logistic regression as appropriate.

R^2 ^for logistic regression is Nagelkerke generalised R

^a ^log-transformed

^b ^Yes vs. No for Parents owned house; Parents owned car; and Reported being bullied

^c ^Non manual vs. Manual for Fathers occupational category

^d ^Tenant vs. Owner occupier for current (adult) home status

* 0.01 ≥ p < 0.05; ** 0.001 ≥ p < 0.01; *** p < 0.001.

As reported in detail elsewhere [37], biomarkers of chronic inflammation (CRP, IL-6) were higher in the more deprived group as were markers of endothelial activation (ICAM and vWF) (Table 1). Likewise, insulin resistance status as assessed by the HOMA-IR score was significantly different between affluent and deprived groups. Carotid atherosclerosis (mean carotid intima-media thickness (cIMT) and the number of participants with plaque present) was more evident in the deprived than the affluent group, despite the fact that observed total cholesterol levels were higher in the latter.

Subjects recruited from deprived areas performed less well in tests of memory recall (Auditory Verbal Learning Test (AVLT)) and executive cognitive function (Stroop test gave a reduced number of correct responses; the "thinking time" component of Choice Reaction Time (CRT) was increased. Their lung function (FEV1) was also poorer.

### Early life conditions and biomarkers of chronic disease

We explored the possibility that variation in inflammatory status, endothelial activation and insulin resistance in adults was related to early life conditions, and then went on to check if the selected health outcomes were associated with early life adversity and the putative intermediary phenotypes (increased chronic inflammation, enhanced endothelial activation and increased insulin resistance).

Relationships (adjusted only for age and sex) between childhood conditions and indicators of potential ill-health in adulthood were assessed by examining the statistical associations of leg length, number of siblings, people/room in the parental home, parental home status and father's occupational category (grouped as non-manual or manual) with phenotypes of increased chronic inflammation, poorer cognitive performance, decreased lung function, prevalence of classical CHD risk factors and carotid atherosclerosis (Table 3). Biomarkers of inflammation and endothelial activation appeared to be influenced little by the number of siblings, moderately by leg length and strongly by early life home conditions and father's occupational category. Likewise, lung function and cognitive performance in adults also appeared to be influenced significantly (P < 0.001) by father's occupation, whether the parents/guardians were owner-occupiers or tenants, and by degree of overcrowding. Cognitive performance was associated also with the number of siblings. Insulin resistance was linked to father's occupational category and whether the participant's parents owned their own home. Carotid IMT was modestly related to leg length but not to home conditions or number of siblings whereas the presence of carotid plaque was related strongly to father's occupation and parental home status, and moderately to the number of people per room and the number of siblings.

**Table 3:** Association of early life conditions with biomarkers of intermediary phenotypes and health outcomes in adulthood

	Quartile of leg length^c ^(shortest to longest)	Number of siblings^d ^0-1; 2; 3; ≥ 4	People per room^e ^≤ 1; > 1, ≤ 2; > 2	Parents owned home^f ^Yes/No	Father's occupation^g ^Non-manual/Manual
***A. Inflammatory and CHD Biomarkers***					
CRP (mg/l)^b^	2.10, 1.57, 1.31, 1.24**	1.43, 1.35, 1.57, 1.70	1.24, 1.64, 2.14***	1.16, 1.71***	1.15, 1.86***
IL-6 (pg/ml)^b^	1.88, 1.69, 1.64, 1.44*	1.80, 1.58, 1.60, 1.71	1.49, 1.72, 1.95**	1.36, 1.80***	1.36, 1.85***
ICAM (ng/ml)^b^	279, 276, 258, 250*	257, 252, 266, 279**	248, 272, 299***	239, 277***	246, 278***
vWF (IU/dl)^a^	144, 140, 143, 133	136, 136, 143, 145	133, 145, 147**	131, 145***	132, 147***
LDL Cholesterol (mmol/l)^a^	2.91, 3.04, 3.03, 3.05	2.99, 3.05, 3.10, 2.93	3.09, 3.00, 2.84	3.13, 2.96*	3.08, 3.02
BP systolic (mmHg)^a^	140, 134, 134, 134*	137, 136, 135, 135	136, 136, 135	133, 136*	135, 136
HOMA-IR^a^	1.70, 1.66, 1.61, 1.60	1.79, 1.60, 1.61, 1.69	1.60, 1.66, 1.78	1.43, 1.75*	1.47, 1.78*

***B. Adult Health Outcomes***					
Stroop test (number correct)^a^	95.7, 97.4, 99.6, 98.9	100.4, 99.9, 98.8, 94.7*	101.6, 96.6, 92.5***	102.9, 95.9***	103.1, 95.5***
Choice Reaction Time (ms)^b^	588, 568, 561, 554	554, 535, 570, 584***	540, 586, 665***	525, 578***	530, 581***
AVLT (words recalled)^a^	11.3, 11.7, 11.7, 12.2*	11.9, 12.1, 11.5, 11.4**	12.0, 11.1, 11.0***	12.2, 11.5***	12.3, 11.3***
FEV1 (L)^a^	2.62, 2.79, 2.95, 3.26***	2.97, 2.97, 2.95, 2.79*	3.11, 2.85, 2.53***	3.19, 2.80***	3.16, 2.78***
Carotid IMT (mm)^a^	0.72, 0.69, 0.71, 0.67*	0.68, 0.69, 0.69, 0.70	0.69, 0.69, 0.72	0.69, 0.70	0.68, 0.70
Plaque present (%)^h^	53.4%, 46.2%, 55.7%, 44.2%	45.1%, 44.5%, 49.6%, 58.7%*	45.4%, 51.7%, 62.8%*	39.6%, 55.2%***	39.9%, 57.2%***

^a ^Mean values for continuous variables adjusted for age and sex;

^b ^Geometric means adjusted for age and sex;

^c ^Entire group of 666 subjects was divided by quartile of leg length (mean length in quartiles 1 through to 4 was 73.0, 78.1, 82.3, 88.2 cm respectively);

^d ^In a similar manner the entire population were divided by number of siblings (0-1, n = 94; 2, n = 176; 3, n = 167; 4 +, n = 227)

^e ^Number of people per room was calculated by dividing the total number of people in the house (adults and children) by the number of rooms (0-1, n = 241; 1-2, n = 342; 2 +, n = 81)

^f ^Owner, n = 188; Not owner, n = 476

^g ^Non-Manual, n = 233; Manual, n = 370

^h ^Percentage values adjusted for age and sex

* 0.01 ≥ p < 0.05; ** 0.001 ≥ p < 0.01; *** p < 0.001.

**Figure 1:**
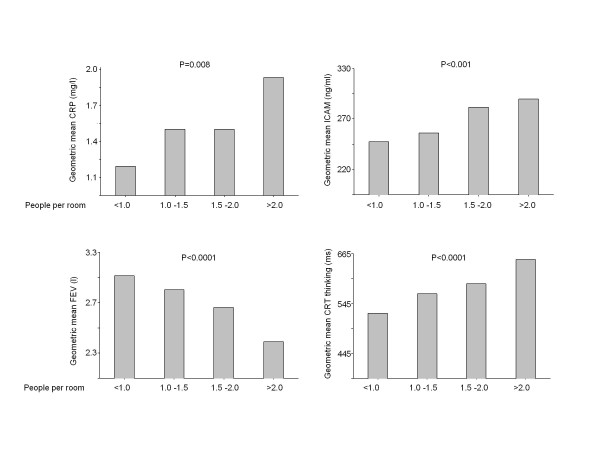
**Influence of early life overcrowding on inflammation, lung function and cognitive performance in adulthood. **The entire group of 666 participants was divided into categories dependent on the number of people (adults and children) in the home when subject was aged 11 years divided by the number of rooms in the home. 241 participants were in the category people/room < 1, 205 participants were in the category people/room 1.0-1.5, 137 participants were in the category people/room 1.5-2.0, and 81 participants were in the category people/room >2. 2 participants did not report the number of rooms in the childhood home. P value is the significance of number of people per room as a predictor of CRP, ICAM, FEV1 and Choice Reaction Time in age and sex adjusted regression models. The height of the bar represents the geometric mean within each category of people/room.

Figure 1 presents the association of overcrowding in the childhood home to biomarkers of chronic disease in adult life. It can be seen that indices of inflammation and endothelial activation (CRP, ICAM) in adulthood were related significantly in an apparently linear fashion to overcrowding in childhood, as were lung function (FEV1) and cognitive function (as assessed by Choice Reaction Time). LDL cholesterol, insulin resistance and blood pressure (data not shown) on the other hand were not.

Since a number of early life conditions were related to inflammatory biomarkers in adults (Table 3), and we postulated that intermediary phenotypes such as chronic inflammation may help explain a range of poor health outcomes in deprived communities, multivariate models were constructed to examine whether the relationship between parental home status, father's occupational category, overcrowding, number of siblings and leg and trunk length contributed independently as predictors of chronic inflammation and endothelial activation in adults (Table 4). It can be seen in Model 1 that father's occupational category remained a predictor of CRP, IL-6 and ICAM when other early life variables were taken into account. Likewise parental home status was a significant predictor of IL-6 and ICAM, while overcrowding was independently associated with ICAM. Family size by itself did not appear to be an important factor. It should be noted, however, that father's occupation, parental home status and overcrowding were highly correlated (Table 2) and these variables may be considered as reflecting general childhood home circumstances at age 11 and are therefore interchangeable in these multivariate analyses. For example in models (not shown) where father's occupation was omitted, parental home status was an independent predictor of all four biomarkers, and overcrowding predicted independently CRP (*P *= 0.031) and ICAM (*P *= 0.00057). To assess the aggregate effect of early life conditions an F-test was performed comparing the overall fit of Model 1 against a base model (Model 0) which included only age and sex. It can be seen for each biomarker that 'early life conditions' did add significantly to the explanatory power of the model (Table 4).

**Table 4:** Multivariate analyses of early life determinants of chronic inflammation and endothelial activation

	log(CRP) β (95% CI)	vWF β (95% CI)	log(IL-6) β (95% CI)	log(ICAM) β (95% CI)
**Model 0^a^**				
Age	0.118 (0.056, 0.180)***	7.874 (5.405, 10.344)***	0.103 (0.066, 0.139)***	0.017 (0.002, 0.032)*
Sex	0.150 (-0.054, 0.355)	-0.964 (-9.060, 7.133)	0.020 (-0.101, 0.142)	0.008 (-0.042, 0.058)
**R^2^**	3.5%	8.2%	6.3%	1.1%

**Model 1^b^**				
Age	0.090 (0.026, 0.154)**	6.694 (4.090, 9.298)***	0.081 (0.043, 0.118)***	0.007 (-0.008, 0.022)
Sex	-0.094 (-0.390, 0.202)	-5.890 (-17.802, 6.021)	-0.187 (-0.360, -0.014)*	-0.063 (-0.133, 0.007)
Parental home status	0.039 (-0.220, 0.298)	9.285 (-1.018, 19.588)	0.159 (0.007, 0.311)*	0.063 (0.001, 0.124)*
Fathers occupation cat.	0.357 (0.118, 0.595)**	7.024 (-2.465, 16.513)	0.181 (0.041, 0.321)*	0.065 (0.008, 0.121)*
People/room	0.109 (-0.041, 0.259)	3.500 (-2.593, 9.593)	0.029 (-0.060, 0.117)	0.042 (0.006, 0.077)*
Number of siblings	-0.026 (-0.098, 0.046)	-0.356 (-3.265, 2.553)	-0.012 (-0.054, 0.030)	0.008 (-0.009, 0.026)
Leg length	-0.019 (-0.041, 0.003)	0.059 (-0.819, 0.936)	-0.013 (-0.026, 0.000)	-0.005 (-0.010, 0.001)
Trunk length	-0.015 (-0.041, 0.012)	-0.680 (-1.746, 0.386)	-0.016 (-0.031, -0.001)*	-0.005 (-0.011, 0.002)
**R^2^**	9.6%	12.1%	14.5%	12.9%
**F-Test vs. Model 0**	p = 0.0001	p = 0.0040	p < 0.0001	p < 0.0001

**Model 2^c^**				
Age	0.017 (-0.035, 0.069)	5.730 (3.157, 8.304)***		
Sex	0.113 (-0.125, 0.350)	-2.014 (-13.639, 9.610)		
Parental home status	-0.153 (-0.361, 0.055)	6.127 (-3.919, 16.174)		
Fathers occupation cat.	0.153 (-0.039, 0.345)	3.676 (-5.598, 12.950)		
People/room	0.069 (-0.052, 0.190)	1.950 (-3.997, 7.898)		
Number of siblings	-0.017 (-0.075, 0.041)	-0.449 (-3.273, 2.375)		
Leg length	-0.006 (-0.024, 0.012)	0.295 (-0.560, 1.149)		
Trunk length	0.003 (-0.019, 0.024)	-0.372 (-1.410, 0.667)		
ICAM	0.476 (0.136, 0.817)**	29.381 (12.536, 46.227)***		
IL-6	0.950 (0.809, 1.090)***	9.051 (2.296, 15.806)**		
**R^2^**	42.7%	17.9%		
**F-Test vs. Model 1**	p < 0.0001	p < 0.0001		
**F-Test omitting Early Life variables**	p = 0.50	p = 0.42		

**Model 3^d^**				
Age	0.081 (0.016, 0.146)*	5.626 (3.028, 8.224)***	0.078 (0.040, 0.116)***	0.007 (-0.006, 0.020)
Sex	-0.011 (-0.306, 0.283)	-3.714 (-15.416, 7.988)	-0.115 (-0.286, 0.056)	-0.005 (-0.066, 0.056)
Parental home status	-0.101 (-0.359, 0.158)	3.579 (-6.584, 13.741)	0.091 (-0.059, 0.242)	0.014 (-0.040, 0.067)
Fathers occupation cat.	0.261 (0.023, 0.499)*	2.130 (-7.225, 11.486)	0.137 (-0.002, 0.276)	0.012 (-0.037, 0.061)
People/room	0.049 (-0.099, 0.197)	1.004 (-4.945, 6.952)	0.002 (-0.085, 0.089)	0.021 (-0.010, 0.051)
Number of siblings	-0.048 (-0.120, 0.023)	-1.182 (-4.014, 1.650)	-0.029 (-0.070, 0.013)	-0.002 (-0.017, 0.013)
Leg length	-0.015 (-0.037, 0.006)	0.225 (-0.623, 1.073)	-0.010 (-0.023, 0.002)	-0.002 (-0.006, 0.002)
Trunk length	0.003 (-0.023, 0.030)	0.209 (-0.862, 1.279)	-0.004 (-0.020, 0.011)	0.005 (-0.001, 0.010)
Current smoker	-0.047 (-0.318, 0.225)	5.318 (-5.522, 16.158)	0.128 (-0.030, 0.287)	0.228 (0.173, 0.283)***
Years of Education	-0.024 (-0.057, 0.009)	-0.950 (-2.192, 0.292)	0.009 (-0.010, 0.027)	-0.005 (-0.011, 0.002)
Current home status	0.340 (0.038, 0.642)*	-2.372 (-14.249, 9.506)	0.171 (-0.004, 0.347)	0.041 (-0.020, 0.103)
Current income^e^	-0.080 (-0.174, 0.013)	-7.379 (-11.045, -3.712)***	-0.068 (-0.123, -0.014)*	-0.041 (-0.060, -0.022)***
**R^2^**	14.8%	19.1%	20.2%	37.8%
**F-Test vs. Model 1**	p < 0.0001	p < 0.0001	p < 0.0001	p < 0.0001
**F-Test omitting Early Life variables**	p = 0.1984	p = 0.9038	p = 0.0452	p = 0.2817

^a ^Model 0 includes age (coefficient calculated as per 5 years) and sex (Male vs. Female);

^b ^Model 1 examined the influence of the early life variables identified as most strongly linked to inflammation and tested their independence;

^c ^Model 2 includes not only early life variables but also intermediary phenotype biomarkers (ICAM and IL-6);

^d ^Model 3 includes early life variables and adult markers of socioeconomic status e.g. years of education, current income and current home status (owner occupier/tenant);

^e ^Regression coefficient calculated as per £10,000 increase in income;

* 0.01 ≥ p < 0.05; ** 0.001 ≥ p < 0.01; *** p < 0.001.

Model 2 in Table 4 was an expanded model which tested whether the associations between early life variables and CRP and vWF persisted when the alternative biomarkers IL-6 and ICAM were included. It can be seen that the association of father's occupation with CRP lost significance when ICAM and IL-6 were entered into the model, and that in aggregate early life variables did not add to the goodness of fit of the model (F-test for Model 2 versus a model with only age, sex, ICAM and IL-6 was not significant).

Table 5 explores in multivariate models the independence of the associations of early life variables with a range of health outcomes related to lung function, cognitive performance and carotid atherosclerosis. It can be seen that father's occupational category and overcrowding were related in Model 1 (Table 5) to FEV1. Father's occupational category was also related to Stroop performance and plaque presence. Leg and trunk length were related independently to FEV1 and measures of cognitive performance. In these age and sex adjusted models, early life variables explained 10.8% to 67% of the variation in the health outcomes. Again, in models where father's occupational category was omitted parental home status became a significant predicator of FEV1 and cognitive function (data not shown). Comparison of goodness of fit with an F-test versus a base model (Model 0) adjusted for age and sex only indicated significant improvement for all five outcomes when general childhood home circumstances were included.

**Table 5:** Multivariate analyses of early life determinants of chronic inflammation and endothelial activation

	FEV_1 _β (95% CI)	log(CRT, Thinking) β (95% CI)	STROOP β (95% CI)	AVLT β (95% CI)	Plaque Presence^f ^OR (95% CI)
**Model 0^a^**					
Age	-0.186 (-0.221, -0.152)***	0.055 (0.044, 0.067)***	-2.98 (-4.00, -1.96)***	-0.259 (-0.382, -0.137)***	1.53 (1.35, 1.74)***
Sex	-0.957 (-1.072, -0.843)***	0.042 (0.003, 0.080)*	1.12 (-2.19, 4.43)	0.497 (0.092, 0.902)*	0.55 (0.37, 0.82)**
**R^2^**	48.6%	17.0%	7.4%	5.0%	15.9%

**Model 1^b^**					
Age	-0.144 (-0.173, -0.114)***	0.047 (0.035, 0.060)***	-2.72 (-3.77, -1.67)***	-0.195 (-0.322, -0.067)**	1.51 (1.32, 1.74)***
Sex	-0.358 (-0.495, -0.221)***	-0.009 (-0.065, 0.047)	1.73 (-3.11, 6.58)	1.102 (0.512, 1.691)***	0.34 (0.18, 0.65)**
Parental home status	-0.072 (-0.191, 0.046)	0.020 (-0.029, 0.069)	-2.07 (-6.27, 2.12)	-0.301 (-0.813, 0.210)	1.29 (0.76, 2.19)
Fathers occupation cat.	-0.135 (-0.244, -0.026)*	0.036 (-0.009, 0.081)	-5.62 (-9.50, -1.74)**	-0.436 (-0.908, 0.036)	1.81 (1.11, 2.94)*
People/room	-0.082 (-0.153, -0.010)*	0.023 (-0.005, 0.051)	-0.10 (-2.59, 2.39)	-0.083 (-0.382, 0.215)	1.05 (0.76, 1.46)
Number of siblings	-0.023 (-0.058, 0.011)	0.006 (-0.007, 0.020)	-0.46 (-1.63, 0.70)	-0.076 (-0.220, 0.068)	1.08 (0.93, 1.26)
Leg length	0.034 (0.024, 0.044)***	0.001 (-0.003, 0.005)	-0.41 (-0.78, -0.05)*	0.015 (-0.028, 0.059)	0.98 (0.93, 1.02)
Trunk length	0.051 (0.039, 0.063)***	-0.008 (-0.013, -0.003)**	0.51 (0.06, 0.95)*	0.069 (0.016, 0.121)*	0.97 (0.91, 1.02)
**R^2^**	67.0%	22.7%	13.0%	10.8%	22.0%
**F-Test vs. Model 0**	p < 0.0001	p < 0.0001	p = 0.0002	p = 0.0001	p = 0.0006

**Model 2^c^**					
Age	-0.133 (-0.163, -0.104)***	0.044 (0.032, 0.057)***	-2.804 (-3.871, -1.737)***	-0.194 (-0.322, -0.066)**	1.53 (1.33, 1.77)***
Sex	-0.400 (-0.534, -0.266)***	0.003 (-0.053, 0.059)	1.504 (-3.350, 6.358)	1.009 (0.425, 1.594)***	0.34 (0.18, 0.65)**
Parental home status	-0.028 (-0.144, 0.088)	0.009 (-0.040, 0.057)	-1.738 (-5.948, 2.472)	-0.212 (-0.719, 0.295)	1.30 (0.76, 2.22)
Fathers occupation cat.	-0.093 (-0.200, 0.014)	0.024 (-0.021, 0.069)	-5.330 (-9.228, -1.432)**	-0.349 (-0.818, 0.120)	1.82 (1.11, 2.99)*
People/room	-0.070 (-0.139, 0.000)	0.019 (-0.010, 0.047)	0.241 (-2.251, 2.733)	-0.016 (-0.312, 0.279)	1.04 (0.74, 1.45)
Number of siblings	-0.020 (-0.054, 0.014)	0.006 (-0.007, 0.020)	-0.352 (-1.513, 0.808)	-0.059 (-0.201, 0.083)	1.08 (0.93, 1.26)
Leg length	0.032 (0.022, 0.042)***	0.002 (-0.002, 0.006)	-0.436 (-0.804, -0.067)*	0.009 (-0.034, 0.052)	0.98 (0.93, 1.02)
Trunk length	0.048 (0.036, 0.060)***	-0.007 (-0.012, -0.002)**	0.498 (0.052, 0.944)*	0.062 (0.010, 0.114)*	0.96 (0.91, 1.02)
ICAM	-0.334 (-0.524, -0.145)***	0.088 (0.008, 0.167)*	-8.734 (-15.589, -1.879)*	-1.674 (-2.505, -0.842)***	1.43 (0.59, 3.48)
IL-6	-0.096 (-0.174, -0.017)*	0.032 (0.000, 0.064)	1.428 (-1.382, 4.237)	0.118 (-0.219, 0.455)	0.84 (0.59, 1.21)
**R^2^**	69.1%	25.1%	14.3%	14.0%	22.3%
**F-Test vs. Model 1**	p < 0.0001	p = 0.0011	p = 0.0444	p = 0.0003	p = 0.5709
**F-Test omit Early Life variables**	p < 0.0001	p = 0.0073	p = 0.0017	p = 0.0199	p = 0.0021

**Model 3^d^**					
Age	-0.147 (-0.177, -0.118)***	0.046 (0.034, 0.058)***	-2.59 (-3.63, -1.55)***	-0.155 (-0.282, -0.027)*	1.58 (1.36, 1.84)***
Sex	-0.412 (-0.545, -0.279)***	0.011 (-0.044, 0.066)	0.07 (-4.67, 4.80)	0.972 (0.392, 1.551)**	0.37 (0.19, 0.72)**
Parental home status	-0.027 (-0.143, 0.089)	-0.011 (-0.059, 0.037)	0.17 (-3.96, 4.29)	0.018 (-0.488, 0.524)	1.19 (0.68, 2.08)
Fathers occupation cat.	-0.081 (-0.189, 0.026)	0.017 (-0.027, 0.061)	-3.80 (-7.63, 0.03)	-0.186 (-0.652, 0.280)	1.57 (0.94, 2.61)
People/room	-0.060 (-0.130, 0.009)	0.010 (-0.017, 0.038)	0.87 (-1.56, 3.29)	0.055 (-0.237, 0.347)	0.99 (0.71, 1.39)
Number of siblings	-0.012 (-0.045, 0.022)	0.001 (-0.012, 0.014)	0.04 (-1.09, 1.17)	-0.029 (-0.169, 0.111)	1.07 (0.92, 1.25)
Leg length	0.032 (0.023, 0.042)***	0.002 (-0.002, 0.006)	-0.46 (-0.81, -0.10)*	0.007 (-0.035, 0.049)	0.98 (0.93, 1.03)
Trunk length	0.042 (0.030, 0.054)***	-0.004 (-0.009, 0.001)	0.16 (-0.29, 0.60)	0.026 (-0.026, 0.079)	0.98 (0.92, 1.04)
Current smoker	-0.232 (-0.356, -0.108)***	-0.008 (-0.058, 0.043)	0.51 (-3.83, 4.85)	-0.051 (-0.587, 0.486)	1.53 (0.85, 2.78)
Years of Education	0.008 (-0.007, 0.023)	-0.003 (-0.009, 0.003)	0.09 (-0.41, 0.59)	0.062 (0.000, 0.123)	0.91 (0.85, 0.98)*
Current home status	-0.115 (-0.253, 0.023)	0.089 (0.033, 0.145)**	-7.09 (-11.94, -2.24)**	-0.287 (-0.877, 0.302)	1.44 (0.75, 2.77)
Current income^e^	0.016 (-0.027, 0.059)	-0.018 (-0.035, 0.000)*	1.81 (0.32, 3.31)*	0.283 (0.101, 0.465)**	1.18 (0.97, 1.44)
**R^2^**	70.0%	29.0%	20.2%	17.6%	25.1%
**F-Test vs. Model 1**	p < 0.0001	p < 0.0001	p < 0.0001	p < 0.0001	p = 0.0128
**F-Test omit Early Life variables**	p < 0.0001	p = 0.6751	p = 0.0941	p = 0.9207	p = 0.2018

^a ^Model 0 includes age (coefficient calculated as per 5 years) and sex (Male vs. Female);

^b ^Model 1 examined the influence of the early life variables identified as most strongly linked to inflammation and tested their independence;

^c ^Model 2 includes not only early life variables but also intermediary phenotype biomarkers (ICAM and IL-6);

^d ^Model 3 includes early life variables and adult markers of socioeconomic status e.g. years of education, current income and current home status (owner occupier/tenant);

^e ^Regression coefficient calculated as per £10,000 increase in income;

^f ^Effect estimates for plaque presence reported as odds ratios; R^2 ^values are Nagelkerke pseudo-R^2^; between model comparisons are likelihood ratio tests;

* 0.01 ≥ p < 0.05; ** 0.001 ≥ p < 0.01; *** p < 0.001.

Model 2 in Table 5 included key biomarkers of the putative intermediary phenotypes (ICAM and IL-6) in the 'early life model' and it can be seen that childhood home conditions were no longer independent predictors of FEV1 although leg and trunk length remained significant, and father's occupational category continued to be a predictor of Stroop performance and of plaque presence. Inclusion of ICAM and IL-6 in the model improved the goodness of fit for FEV1, CRT, Stroop and AVLT (significant F-test for Model 2 versus Model 1). Conversely, early life variables contributed also in aggregate in that the F-test was significant for all outcomes when Model 2 was compared to a model with only age, sex, ICAM and IL-6.

### Impact of contemporary indicators of socioeconomic status

In multivariate models (Table 4, Model 3) which included indicators of individual socioeconomic status (education, income, current home status) as adults, early life conditions were overall less important as predictors of IL-6, and ICAM, although for CRP father's occupational category remained significant. Average household income and current home status, and for ICAM current smoking, were related independently to these individual biomarkers. Early life variables could be omitted from Model 3 without significant change in the goodness of fit for CRP, vWF and ICAM. However, they did appear to contribute significantly to the IL-6 model (F-test = 0.045).

A similar phenomenon can be seen in Table 5 (Model 3) where childhood home circumstances identified as predictors in Model 1 were no longer associated significantly with measures of lung function, cognitive performance and carotid atherosclerosis. Leg length and trunk length, however, remained significant in models for FEV1 and performance on the Stroop test. In Model 3 when early life variables were omitted the goodness of fit for FEV1 was reduced (F-test p < 0.0001), but these variables did not contribute significantly to models of other health outcomes when adult SES was included.

## Discussion

Chronic inflammation is considered to be a 'common soil' in the aetiology of a number of diseases and disorders including cardiovascular disease and type 2 diabetes [45]. It also appears to be related to cognitive decline in older people [21-23]. This study explored possible links between early life adversity, intermediary phenotypes, and a range of poorer health outcomes in deprived communities. By examining the statistical associations between variables we found evidence that childhood living conditions may impact on the state of activation of the innate immune system and on endothelial activation in adult life. Notably, father's occupational category, whether or not the subject's parents owned the family home, and a measure of overcrowding in the home (number of occupants divided by number of rooms) showed significant associations with biomarkers of inflammation and endothelial dysfunction. The relationships were independent of the number of children in the family and of leg length (as an index of nutrition and growth) [31-33] and trunk length (as an index of chronic childhood illness [32]). These findings add weight to the postulate that the social and family environment in early life influences through biological pathways the propensity to develop common, chronic diseases in later life. Emerging data also suggest that the duration of childhood spent in poverty or in a household of low socioeconomic status has an effect that accumulates over time to adversely affect morbidity and mortality in later adulthood [46,47].

Indices of lung function, cognitive performance and carotid artery plaque presence appear to be likewise affected by adverse early life conditions. This finding is in line with earlier work showing a prospective association between the duration of childhood poverty and adult working memory; an association which in part appears to be explained by elevated chronic stress during childhood [48]. The observation that inclusion of IL-6 and ICAM in multivariate models (Model 2) reduced the importance of father's occupation/parental home conditions (owner-occupier status and overcrowding) as potential predictors suggests that chronic inflammation and endothelial activation may be intermediary phenotypes in the relationship between adverse childhood home conditions and poorer lung function. The results of the present analysis are in line with a recent report that a cumulative score of socioeconomic position (in childhood, young adult life and midlife) was strongly related to CRP and haemostasis (fibrinogen and tissue plasminogen activator) in adults [14]. Early life socioeconomic status has also been shown recently to be significantly associated with CRP levels, independent of later life socioeconomic status, with adiposity accounting for the majority of this association between life-course socioeconomic indicators and CRP levels [49]. Similarly, it has been reported that adolescent females who spent their early life in a family-owned, as opposed to a rented, home had lower levels of expression of specific inflammatory genes in peripheral blood monocytes [50]. In a systematic review of population-based studies examining CRP levels and indicators of socioeconomic position, race and ethnicity, elevated CRP levels were associated with increasing poverty and non-white race [51]. Similarly, an investigation of the life course association between childhood maltreatment and adult inflammation in a birth cohort as part of the Dunedin Multidisciplinary Health and Development Study, maltreated children showed a significant and graded increase in CRP levels in adulthood [52], providing evidence of a causal association between childhood maltreatment and adult inflammation and evidence of a dose-response relation between severity of maltreatment and inflammation. Low birth weight and infection in childhood are also related to endothelial dysfunction [53] and these may be additional mechanisms by which childhood socioeconomic circumstances relate to these adult biomarkers of chronic disease. However, these association studies cannot eliminate the impact of unmeasured potential confounders on the outcome of interest. Thus, while the induction of chronic inflammation is plausible as a mechanistic application, further work needs to be done to establish cause and effect.

Glasgow over the period when our subjects were children (1950-1980) had substantial areas where housing was poor and overcrowding common [55]. The data provided here indicate that where there was an average of more than 1.0 person per room in the childhood home, the risk of developing elevated concentrations of inflammatory markers in adult life increased. There is previous evidence that overcrowding leads to a greater chance of respiratory infection [55-57] and our data are consistent with the suggestion that the heightened inflammatory response becomes constitutive. The adverse effects, however, appeared to be specific in that overcrowding was not associated with variation in other CHD risk factors such as higher cholesterol concentrations, blood pressure or insulin resistance in adults.

In statistical models that included both early life conditions and contemporary indices of adult socioeconomic status, the latter were clearly more important determinants of IL-6 and ICAM and possibly incorporated most of the predictive information inherent in the early life variables, although father's occupational category persisted as an independent factor for CRP. For FEV1 and CRT, in models which omitted father's occupation, overcrowding in the childhood home remained a significant predictor even when individual level socioeconomic indices were included. The influence of early life conditions on cognitive executive function (that is comparing Models 0 and 1 in Table 5) is consistent with earlier reports of executive dysfunction in children living in deprived circumstances [58,59]. The aetiological links underlying these associations are likely to be complex and include the increased likelihood of childhood illness (and missed education) in overcrowded homes, as well as an increased risk of compromised lung function.

There are limitations inherent in the design of this study. First, the sample was selected from the ends of the Scottish Index of Multiple Deprivation (SIMD) gradient, and therefore does not represent the population as a whole. In 2004 at the time of sampling, 31.4% of the population of Glasgow fell into the bottom 5% of the SIMD classification, and 6% fell into the top 20% of the SIMD. The study reflects the socioeconomic extremes seen in the city but does not provide information on the nature of the gradient of outcome indicators across all SIMD categories, nor is it representative of Scotland's - or Glasgow's - population as a whole. Second, there is possible response bias, particularly due to the difficulties of recruiting younger men from the most deprived areas, although the response rate at about 25% is not unusual for population based surveys [60,61]. To explore the extent of any response bias, we examined the characteristics of non-respondents and found that within each age, sex and socioeconomic stratum participants were comparable broadly to non-participants on the measures available [36,37] (Appendix 1). Third, the early life and childhood conditions of participants at age 11 years were assessed by recall, rather than by objective measures taken historically. Indicators of childhood social class, especially relating to father's occupational social class, may therefore have been wrongly reported by participants being asked to remember information up to five decades later. Further, those with cognitive impairment may have been less accurate in their recall and may have introduced confounding variation into the analysis. Fourth, there is the possibility that some who experienced adverse childhood conditions now live in a middle income neighbourhood and were not included in the sample. The extent to which this influences the overall findings depends on whether the associations between variables present in this absent group are reflected in the approximately 40 to 50% of 'least deprived' individuals who had a less favourable childhood (parents were tenants, did not own a car or father was a manual worker). Finally, the cross-sectional design means that we cannot identify temporal relationships between variables (although these are of course inherent in the relationship between early life and adulthood), and so can only report associations.

## Conclusions

This study highlights potential problems in attempting to redress the imbalance in health between socio-economically affluent and deprived groups, a key concern of governments [2,62,63]. Here, we present evidence that adverse early life conditions are associated with the setting of the innate immune system and with endothelial activation (putative intermediary phenotypes) in adult life. This, in turn, is likely to increase the propensity to develop a range of chronic diseases. Housing investment, area-based regeneration and 'early years' interventions are now common place, yet a small percentage of the population in Scotland (4.6%), and specifically in Glasgow (7.2%), continue to live in overcrowded households [64]. Future interventions need to be based on the best possible evidence about the many complex and inter-related factors that generate and maintain social and health inequalities, and the greatest gains in advancing population health and in reducing health inequalities will, predictably, result from investment to improve social and economic conditions in both early and later life.

## Abbreviations

AVLT: Auditory Verbal Learning Test; CHD: Coronary Heart Disease; CRP: C reactive protein; FEV1: forced expiratory volume in 1 second; ICAM: intercellular adhesion molecule 1; IL-6: interleukin-6; pSoBid: Psychological; Social and Biological determinants of Ill health study; SES: socioeconomic status; vWF: von Willebrand Factor.

## Conflict of interests

The authors declare that they have no competing interests.

## Authors' contributions

CJP, VB, JSM, GDB, IF, HB, JC, KAD, MH, AM, KM, NS, PGS, YNV and CT contributed equally to conception, design and final approval of the version of the manuscript. CJP and JSM have been involved in drafting the manuscript and revising it critically for important intellectual content. VB performed the statistical analysis. YNV and AM supervised the recruitment of subjects and data collection. MH produced the Registrar General Social Classification devised from the textual and quantitative data provided by respondents and commented on drafts of the manuscript. GDB contributed to the conception, design and final approval of this version of the manuscript while on sabbatical at The George Institute for International Health, Sydney, Australia.

## Pre-publication history

The pre-publication history for this paper can be accessed here:

http://www.biomedcentral.com/1471-2458/11/42/prepub

## Supplementary Material

Additional file 1 **Appendix 1 - Comparison of 'participants' versus 'non-participants' using GPASS data on smoking status and prescription medication. **GPASS (General Practice Administration System Scotland) is a software programme widely used by GPs in Scotland to maintain patient health records. The data provided in the table in additional file 1 were extracted (without patient identifiers and with permission) from computers in 8 of the 10 practices involved in the study. The values provided are mean percentages for each characteristic from subjects who were invited to participate and attended Visit 1 ('participants') and those who were invited but did not respond or declined ('non-participants'). Participants (n = 666) and non-participants (n = 1654) were by design drawn from the same age and sex categories. A further 392 non-participants were located in the 2 practices where GPASS was not used and hence we had no further information available (giving a total of 2712 invitees). While there were statistically significant differences between participants and non-participants in certain characteristics, these did not appear to be of a magnitude to suggest that the associations seen in study participants were not representative of the population sub group from which they were drawn. Since we have no detailed data from non-participants, this assumption cannot be tested rigorously and selection bias is a potential limitation in the interpretation of the study findings. Click here for file
