# Supplementary material for: Early life socioeconomic adversity is associated in adult life with chronic inflammation, carotid atherosclerosis, poorer lung function and decreased cognitive performance: a cross-sectional, population-based study
Source: BMC Public Health. 2011 Jan 17;11:42. doi: 10.1186/1471-2458-11-42 (PMC3032683; doi:10.1186/1471-2458-11-42)
Supplement: Additional file 1 — Appendix 1 - Comparison of 'participants' versus 'non-participants' using GPASS data on smoking status and prescription medication. GPASS (General Practice Administration System Scotland) is a software programme widely used by GPs in Scotland to maintain patient health records. The data provided in the table in additional file 1 were extracted (without patient identifiers and with permission) from computers in 8 of the 10 practices involved in the study. The values provided are mean percentages for each characteristic from subjects who were invited to participate and attended Visit 1 ('participants') and those who were invited but did not respond or declined ('non-participants'). Participants (n = 666) and non-participants (n = 1654) were by design drawn from the same age and sex categories. A further 392 non-participants were located in the 2 practices where GPASS was not used and hence we had no further information available (giving a total of 2712 invitees). While there were statistically significant differences between participants and non-participants in certain characteristics, these did not appear to be of a magnitude to suggest that the associations seen in study participants were not representative of the population sub group from which they were drawn. Since we have no detailed data from non-participants, this assumption cannot be tested rigorously and selection bias is a potential limitation in the interpretation of the study findings. [file 1471-2458-11-42-S1.DOC]

**Additional file**

Appendix 1: Comparison of ‘participants’ versus ‘non-participants’ using GPASS data on smoking status and prescription medication.
